# A randomised controlled demonstration trial of multifaceted nutritional intervention and or probiotics: the healthy mums and babies (HUMBA) trial

**DOI:** 10.1186/s12884-016-1149-8

**Published:** 2016-11-24

**Authors:** Karaponi Okesene-Gafa, Minglan Li, Rennae S. Taylor, John M. D. Thompson, Caroline A. Crowther, Christopher J. D. McKinlay, Lesley M. E. McCowan

**Affiliations:** 1 Department of Obstetrics and Gynaecology, Faculty of Medical and Health Science, University of Auckland, Private Bag 92019, Auckland, New Zealand; 2 South Auckland Clinical School, Faculty of Medical and Health Science, The University of Auckland, Auckland, New Zealand; 3 The Liggins Institute, The University of Auckland, Auckland, New Zealand; 4 Department of Paediatrics, Child and Youth Health, Faculty of Medical and Health Sciences, The University of Auckland, Auckland, New Zealand

**Keywords:** Study protocol, Obesity, Nutritional intervention, Randomised controlled trial, Gestational weight gain, Probiotics, Birthweight

## Abstract

**Background:**

Maternal obesity is associated with adverse pregnancy outcomes and has lifelong negative implications for offspring health. The Institute of Medicine recommends limited gestational weight gain (GWG) in obese women for optimal maternal and infant outcomes. However, there is a gap regarding an effective and sustainable intervention strategy to achieve this goal. The aim of the healthy mums and babies (HUMBA) demonstration trial is to assess whether a multifaceted nutritional intervention and/or an oral probiotic treatment in obese pregnant women can reduce excessive GWG and optimise pregnancy outcomes.

**Methods and design:**

The study is a two by two factorial randomised controlled demonstration trial conducted in Counties Manukau health region, New Zealand, a multi-ethnic region with a high prevalence of obesity. A total of 220 non-diabetic obese women with a singleton pregnancy will be recruited between 12^0^ and 17^6^ weeks. At recruitment, women are randomised to receive either a culturally tailored multifaceted dietary intervention or routine dietary advice, and either an oral probiotic or placebo capsule. Randomisation is undertaken via a web-based protocol, randomize.net, with a 1:1 ratio using stratification by body mass index (BMI) category (BMI of 30–34.9 or BMI ≥35 kg/m^2^). The dietary intervention includes 4 customised nutrition education visits by a trained community health worker combined with motivational text messaging. Probiotic capsules consist of *Lactobacillus rhamnosus GG* and *Bifidobacterium lactis BB12* at a dose of 7 × 10^9^ colony-forming units one per day until birth. Probiotic and placebo capsules are identically pre-packed and labelled by a third party, and are prescribed in a double blinded fashion. Research assessments are conducted at enrolment, 28 weeks, 36 weeks, at birth and at 5 months post-delivery. The primary outcomes for the study are proportion of women with excessive GWG and infant birthweight.

**Discussion:**

The HUMBA demonstration trial will assess the efficacy of a culturally tailored multifaceted dietary intervention and probiotic treatment in limiting excessive GWG and optimising birthweight in a multiethnic sample of obese pregnant women. If successful, either one or both of the interventions may be incorporated into future studies powered to investigate important pregnancy outcomes.

**Trial registration:**

Australian New Zealand Clinical Trials Registry registration number: ACTRN12615000400561, Universal Trial Number: U1111-1155-0409. Date registered: 29^th^ April 2015.

## Background

The global obesity epidemic is of increasing concern, with 671 million people in the world currently estimated to be obese (body mass index (BMI) ≥ 30 kg/m^2^) [1]. Data from the United States suggests that without effective interventions, the rise in obesity will soon lead to reduction of life-expectancy in high-income countries [2]. New Zealand was rated the third most obese nation in the Organisation for Economic Co-operation and Development countries in 2014 [3]. The latest New Zealand national health survey showed that about 30% of women at child-bearing age have a BMI of 30 or more, and the trend is increasing [4]. Of specific concern, the obesity rates are double in Māori and Pacific adults and children compared with those from European/other ethnic backgrounds [4], which highlights ethnic inequalities in the obesity epidemic in New Zealand.

Obese pregnant women have increased rates of most pregnancy complications including gestational diabetes (GDM), pre-eclampsia, caesarean section, and postpartum haemorrhage [5]. Their infants are at higher risk of congenital abnormalities, stillbirth, being born large for gestational age (LGA) and consequential traumatic birth and asphyxia [6, 7]. They are also at increased risk of a range of neonatal complications, including respiratory problems, sepsis, seizures, hypoglycaemia and feeding difficulties [8]. In the long-term, maternal obesity has been associated with lower offspring cognitive function and increased risk of attention deficit hyperactivity and other psychiatric disorders [9, 10]. Moreover, maternal obesity is associated with adverse cardiometabolic health in the offspring. Fetal exposure to excessive nutrients such as maternal hyperlipidaemia and hyperglycaemia, can result in accelerated growth, especially of adipose tissue [7]. Larger infants with increased fat mass are more likely to become obese children [11], and are predisposed to high blood pressure, type 2 diabetes and other metabolic dysfunctions in adulthood [12, 13]. This creates a vicious intergenerational cycle, termed “developmental overnutrition” [14], which may have contributed to the increases in obesity and type 2 diabetes observed over the recent decades.

Based on a number of observational studies, the Institute of Medicine recommends 5–9 kg of gestational weight gain (GWG), in obese women, for optimal maternal and infant outcomes [15]. Exceeding recommended GWG is associated with increased risk of GDM, preeclampsia, LGA, and caesarean in labour independent of maternal BMI [16, 17]. Mothers with excessive weight gain are less likely to lose weight between pregnancies, and may enter further pregnancies even more overweight [18, 19]. Further, excessive GWG compounds the associations between maternal obesity and offspring metabolic dysfunction and cognitive problems [9, 20, 21]. However, implementation of GWG guidelines in practice is challenging. Studies suggest that obese women are more likely to gain an excessive amount of weight during pregnancy than non-obese women [22], and may require more theoretically-designed interventions [23]. There is also an emerging demand from prenatal care providers for effective and reproducible intervention guidance [24].

Lifestyle interventions during pregnancy may limit GWG, however, limited data are available to identify the key components of intervention(s) that are responsible for the positive outcomes [23]. A systematic review reported that amongst diet, physical activity, and mixed approach interventions, dietary interventions were associated with the largest reduction in GWG (4 kg on average) and also with improved pregnancy outcomes [25]. A UK pilot trial of intensive behavioural intervention in obese pregnant women suggested that there is greater potential for change in dietary intake than in physical activity [26]. However, it has been consistently reported from several recent randomised controlled trials (RCT) that lifestyle interventions alone have a limited effect on reducing GWG in obese pregnant women [27–29]. Additional interventions in combination with lifestyle interventions therefore require evaluation in clinical trials to determine whether such combinations can achieve improvements in GWG.

Mobile phone texting technologies are increasingly being used to assist with weight loss. A systematic review has shown that use of mobile phone technologies in non-pregnant populations can result in successful weight loss with increased physical activity and improved nutrition [30]. There was a high level of acceptability and user satisfaction rating, with a significant number of participants stating they would recommend text messaging as a primary intervention to others [30]. A New Zealand multi-ethnic study, in an obese non-pregnant cohort, confirmed the feasibility of using mobile phone technology together with behaviour change techniques. They reported successful weight loss in participants [31]. A recent pilot study of 35 overweight and obese pregnant women reported a 2.7 kg mean GWG reduction with a texting intervention [32].

Modification of the gut microbiome by ingestion of probiotics is a novel pathway for possible intervention to prevent metabolic disease. The microbiome influences energy extraction from food [33], and satiety, inflammation, and glucose and lipid metabolism [34–36], with potential to reduce obesity and type 2 diabetes [37]. Probiotics are safe in pregnancy [38, 39] and provide a simple intervention in pill form. A randomised controlled trial of probiotics/placebo (*Lactobacillus rhamnosus GG* and *Bifidobacterium lactis* at 10^10^ colony-forming units/day) and nutritional advice in pregnant women in Finland showed an over 60% reduction in GDM, with a prevalence of 13% in the probiotic/nutrition group compared with 36% in placebo/nutrition group and 34% in controls [40]. In addition, a reduction in maternal central adiposity at 6 months postpartum and a 127 g average reduction of birthweight were demonstrated in the probiotic treatment group [41, 42]. Besides the reported efficacy of these probiotic capsule in reducing GDM, daily probiotic yoghurt has been associated with a 40% reduction in preeclampsia [43] which is also a common complication in obese pregnant women. A RCT of probiotics/placebo aimed to prevent GDM in overweight and obese women has started in Brisbane, Australia [44], but no studies have been reported to date, confined to obese pregnant women or in combination with intensive dietary intervention, aimed to prevent excessive GWG.

Pregnancy is described as a “teachable moment” providing a finite window during which women are more likely to undergo behavioural change if there are perceived benefits for their offspring [45]. Accordingly, we are undertaking an innovative, randomised controlled demonstration trial of probiotics or placebo plus an intensive, culturally tailored multifaceted dietary intervention in obese pregnant women in South Auckland, New Zealand. We hypothesise that in obese pregnant women the interventions will reduce: 1) the incidence of excessive GWG; and/or 2) infant birthweight.

## Methods and design

### Study design and the setting

The healthy mums and babies (HUMBA) study is a single centre two by two factorial randomised controlled demonstration trial (parallel groups) designed according to CONSORT guidelines [46] and the SPIRIT guidelines for preparation of protocols [47]. We aim to investigate whether or not an oral probiotic capsule consisting of *Lactobacillus rhamnosus GG* and *Bifidobacterium lactis BB12* at a dose of 7 × 10^9^ colony-forming units per day each, or a multifaceted dietary intervention, can reduce excessive GWG and optimise infant birthweight in obese pregnant women.

The HUMBA study is being conducted in the multi-ethic Counties Manukau Health (CMH) region, South Auckland, New Zealand, where 40% of pregnant women are obese in early pregnancy [48]. The ethnic distribution of the birthing population (>7000 per annum) in CMH is 36% Pacific, 24% Māori, 17% Asian, and 23% European/other [48]. There is high socioeconomic deprivation in the region with 53% of the women who birthed in the area in 2013 categorised as being in the lowest socioeconomic quintile for New Zealand [48]. The perinatal mortality is higher in CMH than in any other district health board in the country [49]. The external review of maternity care in CMH has iterated obesity as a major risk factor for stillbirth in the Pacific community [49]. There is an urgent need for effective and sustainable interventions in this high-risk population to improve maternal and child health.

### Inclusion criteria

Women with a singleton pregnancy, BMI ≥ 30 kg/m^2^, between 12^0^ and 17^6^ weeks of gestation and able to provide informed written consent.

### Exclusion criteria

Pre-existing diabetes or HbA1c at booking ≥ 50 mmol/mol [50], taking probiotic supplements, known congenital abnormality, medications or medical conditions which alter glucose metabolism, multiple pregnancy, bariatric surgery, and severe hyperemesis.

### Randomisation

**Fig. 1:**
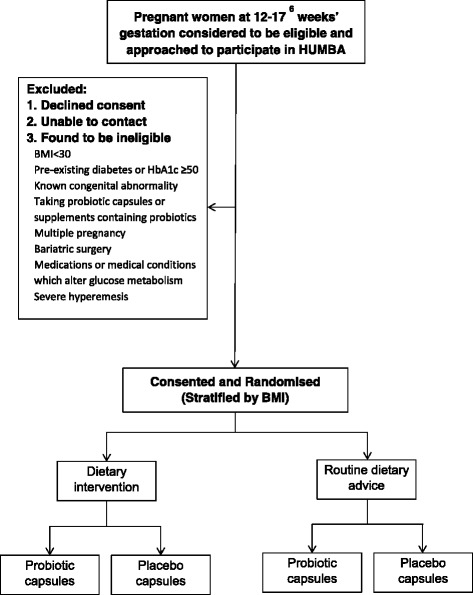
Overview of recruitment and randomisation in the HUMBA study

Randomisation is undertaken using a web-based protocol, randomize.net (http://randomize.net), using random block sizes (minimum 4; maximum 8). For randomisation purposes, each research midwife will serve as a proxy for ‘clinical site’ (this will enable each research midwife to be able to dispense the randomised study capsules at recruitment). Participants are stratified by ‘clinical site’ (n = 2) and BMI category (BMI of 30–34.9 or BMI ≥35 kg/m^2^) and randomly allocated in a 1:1 ratio to dietary intervention or routine dietary advice; and 1:1 ratio to probiotic or placebo (Fig. 1).

### Study interventions

1. Dietary intervention vs routine dietary advice
2. Dietary intervention
  The multifaceted dietary intervention comprises the following four components.
  - Encounters with nutrition advisor
    The nutrition advisors are community health workers (CHW), usually of Pacific or Māori ethnic background, and are experienced in engaging hard-to-reach women in maternity care in our target population. CHW are an integral part of the health workforce in Counties Manukau. They have been trained in the Pacific Heart Beat Certificate of Nutrition by the New Zealand National Heart Foundation, which covers general nutrition as well as complementary extra training in nutrition during pregnancy [51]. A New Zealand registered dietitian has developed a standard operating procedure manual for the CHWs, and provides support, advice and oversight of the nutritional education package administered to participants by the CHWs.
    The encounters include taking an initial brief diet and physical activity history followed by education on appropriate weight gain and in-depth education on healthy eating. The dietary education includes advice about: portion control, healthy food and drink choices, limiting energy dense foods, healthy recipes, label reading and managing cravings.
    Each participant has an initial 1–1.5 h educational session (on average at about 14 weeks’ gestation) with the CHW. Three further 30–60 min face-to-face sessions are planned with the CHW at two weekly intervals and be completed by 28 weeks. Compliance with the dietary intervention is being assessed by the number of educational sessions participants attend with the CHW.
    A HUMBA participant handbook containing detailed information about the suggested content of each of the four dietary education encounter visits is provided to women randomised to the dietary intervention. Participants are also encouraged to use the healthy recipes provided in their handbook.
  b) Behaviour change techniques
    CHWs have received training in evaluation methodology and counselling techniques, including healthy conversations [52]. Behaviour change techniques are incorporated in the nutrition education sessions including identifying barriers, self-monitoring, goal setting, and providing regular feedback [53]. Targets will be set for optimal GWG at the first visit with the CHW. Weight is measured and plotted on a personalised GWG chart, in the participant handbook, at each subsequent encounter.
  c) Physical activity advice
    Physical activity advice which is included in the dietary intervention incorporates information from the Te Wai o Rona program (http://www.sportwaikato.org.nz/resources-library.aspx?resource=document-resource-category-2) [54] and the Royal College of Obstetricians and Gynaecologists (RCOG) guidelines for recreational exercise in pregnancy [55]. In Te Wai o Rona, there are four key physical activity messages which are relevant to pregnant women: 1) look for ways to be active every day, 2) increase daily exercise, 3) move more, add more steps, and 4) reduce sedentary leisure time. The RCOG guideline recommends that previously sedentary women begin with 15 min of exercise focusing on walking three times weekly and gradually increasing to 30 min sessions four times a week to daily [55].
  d) Motivational texting
    Three times weekly motivational texting, which reinforces the educational content covered in the face-to-face meetings with the CHW, is implemented for those participants in the dietary intervention with cell phones (in our recent survey of pregnant women at CMH, 98% had a cell phone [56]). Text messaging continues until birth and incorporate messages about diet and physical activity.

1.2 Routine dietary advice
  These women receive current best practice advice including a pamphlet produced by the New Zealand Ministry of Health (Eating for Healthy Pregnant Women) which contains dietary advice that follows current New Zealand nutrition guidelines [57]. They also receive a pamphlet providing information about healthy weight gain and physical activity in pregnancy [58].

2. Probiotic/placebo intervention
  Probiotic or placebo capsules are taken once daily from enrolment between 12^0^ and 17^6^ weeks of gestation until birth.

2.1 Probiotic intervention
  Participants randomised to probiotic treatment will receive probiotic capsules containing Lactobacillus rhamnosus GG and Bifidobacterium lactis BB12 (Chr. Hansen A/S, Horsholm, Denmark) at a dose of 7 × 109 colony-forming units per day each. Chr. Hansen A/S make the Probio-TecÒ BG-Vcap-6.5 capsules from library colonies which are meticulously DNA fingerprinted to ascertain the presence of Bifidobacterium BB12 and Lactobacillus rhamnosus GG only. The packaging and storage of the probiotics will comply with company specifications ensuring the quality of the product. This is the equivalent probiotic combination that was used by Luoto et al. in the Finnish study [40] and also being used in the ongoing study in Queensland [44].

2.2 Placebo
  Participants randomised to placebo will receive identical capsules containing microcrystalline cellulose and dextrose anhydrate, also supplied by Chr. Hansen A/S, Horsholm, Denmark.

### Allocation concealment and blinding

Christian Hansen has provided identically packaged canisters of placebo and probiotic capsules, containing 31 capsules each. AnQual Laboratories (School of Pharmacy, University of Auckland) have labelled the canisters using a pre-allocated random list. The kit list used to label the canisters was generated by the Project Manager and AnQual, using the Excel random function. This list has secure password protection and is stored with AnQual. The Project manager is the only HUMBA staff member with access to the probiotic/placebo allocation. Although it will not be possible for clinical and research staff to be blinded to the dietary intervention allocation, the key health outcomes including GWG, infant birthweight and results from the oral glucose tolerance test (OGTT), are not subject to bias.

### Assessment of compliance

Compliance with the dietary intervention will be assessed by the number of educational sessions participants attend with the CHW. Compliance with probiotic/placebo will be assessed by the research team via participant self-report at 28 weeks, 36 weeks and birth visits.

### Engagement for recruitment

We have a multi-pronged approach to optimise recruitment including through lead maternity caregivers (self-employed midwives), community antenatal clinics, general practitioners, practice nurses, ultrasound clinics, community contacts, and social media. Our research team provides educational sessions for the health care providers to maximise reach for recruitment. Lead maternity caregivers notify the research team about contact details of eligible women who are interested in participating. The research midwife arranges a time to meet the woman in a suitable location to explain the study, confirm eligibility and obtain informed consent.

In order to exclude women with previously unrecognised Type 2 diabetes, HbA1c is measured in all participants (using the Roche cobas b 101 point-of-care system) prior to randomisation. If HbA1c is ≥50 mmol/mol, women are ineligible and considered to have undiagnosed diabetes [50]. They will be referred to the Diabetes in Pregnancy Service at CMH. If the HUMBA point of care HbA1c is <50 mmol/mol, then the result is not revealed to health practitioners.

### Measures/data collection

**Fig. 2:**
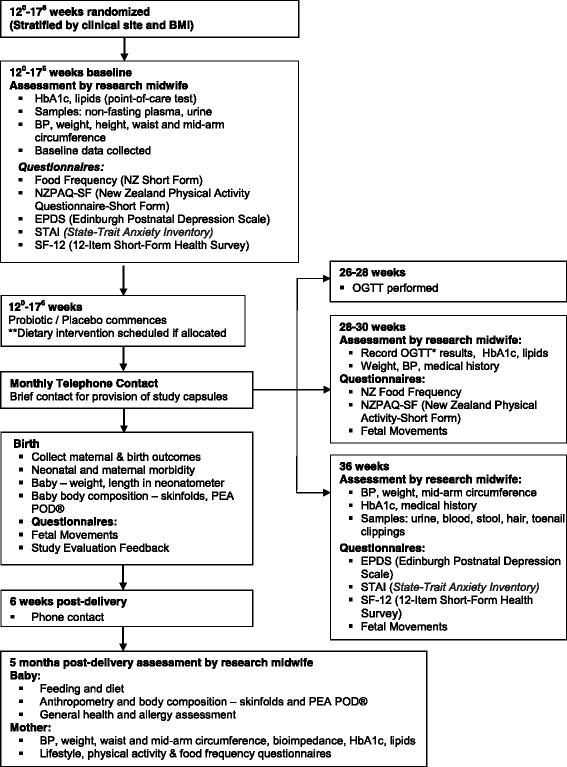
Research plan flow chart for the HUMBA study. * Fasting, one-hour and two-hour glucose after 75 g glucose load. ** Women randomised to the dietary intervention will be seen 4 times at 2–3 weekly intervals between recruitment and the 26–28 week OGTT

Members of the research team will meet participants at pre-specified intervals during pregnancy (Fig. 2) to collect outcome data, provide further supplies of probiotic/placebo and check compliance.

Minimal data are collected with verbal consent in referred eligible women who decline to participate in the study. These minimal data include self-reported weight, height, age, ethnicity and reason for their non-participation.

#### 1st assessment visit (12^0^ – 17^+6^ weeks)

Confirmation of eligibility, informed consent, and randomisation is conducted at the first assessment visit. Comprehensive information is obtained from the woman and her clinical records, including: demographic, socioeconomic, educational and employment data; medical history; obstetric history such as parity, method of conception, gestation and accuracy of estimated date of delivery, previous pregnancy complications (GDM, hypertensive disease, pre-term delivery, caesarean section); family history of diabetes, hypertension and cardiovascular disease; history of smoking, alcohol and other drug use; medications and nutritional supplements; probiotic food ingestion; maternal anthropometrics: weight, height, waist and mid-arm circumference; blood pressure (BP); finger-prick blood lipid testing (Roche cobas b 101 point-of-care system); samples collection : non-fasting blood and urine specimen for biobank; questionnaires including: New Zealand Food Frequency Questionnaire - Short Form [59], New Zealand Physical Activity Questionnaire [60], Edinburgh Postnatal Depression Scale (EPDS) [61], State-Trait Anxiety Inventory (STAI) [62], and the 12-item Short-Form Health Survey (SF-12) [63]. It takes approximately 30 min to complete these questionnaires.

#### 2^nd^ assessment visit (28–30 weeks)

All women in the HUMBA trial are requested to have an OGTT at 26–28 weeks. The second assessment visit is scheduled after the OGTT at approximately 28–30 weeks. Weight, mid-arm circumference and BP are measured. The food frequency, physical activity questionnaires and a fetal movement questionnaire are completed by the participant. The fetal movement questionnaire is adopted from a previous study [64]. An updated medical history is obtained by the research midwife. The OGTT results are reported to the research team with fasting, one- and two-hour results enabling a diagnosis of GDM by the International Association of Diabetes in Pregnancy Study Groups (IADPSG) criteria [65]. The maternity care provider receives fasting and two-hour glucose results from the OGTT as per usual clinical practice using the New Zealand Society for the Study of Diabetes (NZSSD) criteria [66]. Women diagnosed with GDM by the NZSSD criteria will be referred by their maternity care provider to the Diabetes in Pregnancy Service (as per usual practice) and managed according to local guidelines including postpartum testing.

HbA1c and lipid concentrations are measured by finger prick using the Roche cobas b 101 point-of-care system.

#### 3^rd^ assessment visit (36 weeks)

Weight, mid-arm circumference, BP, and HbA1c and lipid concentrations (Roche cobas b 101 point-of-care system) are measured. Any pregnancy complications to date and use of antibiotics are recorded. The fetal movement questionnaire and functional health and well-being questionnaires including EPDS, STAI and SF-12 are repeated. Maternal urine, blood, stool, toenail clippings and hair samples are collected from consenting women for future molecular biology studies.

#### 4^th^ assessment visit/post-delivery evaluation

Maternal and neonatal outcomes are collected within 72 h of birth by a research midwife. Infants are weighed at birth (Mobile electronic baby scales, SECA 334, Germany) and detailed anthropometric measurements obtained by research midwives within 72 h of birth including crown-heel length by neonatometer, head, left mid-arm, chest and abdominal circumferences using a lasso tape, and subscapular, triceps and supra-iliac skinfolds by Harpenden calipers (average of 2 measurements, or median of 3 if initial measurements differ by >0.4 mm) [67]. Among consenting participants, infant body compositions are measured by air displacement plethysmography (PEA POD® Cosmed, Illinois, USA) as soon as practical after birth. In these infants, anthropometry is performed at the same time as the PEA POD® measurement. The rest of the birth outcomes are collected from the mothers and the babies’ clinical records.

A short survey is administered to obtain feedback about participation in HUMBA and the study interventions, and the fetal movement questionnaire is repeated.

#### 5^th^ assessment visit (5 months postpartum)

A follow up appointment with the research midwife and a paediatrician are scheduled at 5 months (±2 weeks) postpartum. Maternal lifestyle and food frequency questionnaires, and infant health and well-being questionnaires are completed. Measurements of maternal BP, weight, height, waist and mid-arm circumference, and body composition by Bioelectrical Impedance Analysis [68] (ImpSFB7®, ImpediMed, Brisbane, Australia) are undertaken. Data are collected on infant feeding, allergies, health and anthropometry (weight and length; head, left mid-arm, chest and abdominal circumference; subscapular, triceps and suprailiac skinfolds), and body composition by PEA POD®, if consented. Infant feeding behaviours are assessed using the Baby Eating Behaviour Questionnaire (BEBQ) [69], and infant eating patterns and nutritional intake will be determined by a food frequency questionnaire. Timing of assessment will be based on corrected age. Consent will be obtained for ongoing contact when funding is obtained for further follow up.

### Primary outcomes

The primary maternal outcome is the proportion of women with excessive GWG, defined as mean weekly weight gain >0.22 kg between recruitment and 36 weeks (or weight at the closest gestation to 36 weeks’ if 36 week weight is unavailable) [15].

The primary infant outcome is infant birthweight.

### Secondary outcomes

Maternal secondary outcomes include:

- Maternal pregnancy glucose metabolism as assessed by OGTT parameters at 26–28 weeks [70], and HbA1c at 28 and 36 weeks and 5 months postpartum.
- Changes in diet quality and dietary patterns between recruitment, 28–30 weeks and 5 months postpartum [59]
- Functional health and well-being (SF-12) at 36 weeks and 5 months postpartum [63]
- Depression and anxiety scores at 36 weeks’ and 5 months postpartum [62, 63]
- Maternal adiposity at 5 months postpartum (assessed by weight, BMI, waist and arm circumference and fat mass measured by bioimpedance)
- GDM by NZSSD criteria [66]
- Pregnancy induced hypertension (preeclampsia and gestational hypertension) [71]
- Mode of birth
- Blood lipid concentrations at 28–30 and 36 weeks’ gestation and 5 months postpartum
- Maternal feedback about participation in the study (survey at post birth visit)

Infant secondary outcomes include:

- Neonatal anthropometry: head circumference and length, and associated Z-scores [72]; birthweight adjusted for length; girths (chest, arm and abdominal) adjusted for length; subscapular, triceps and suprailiac skin fold thickness adjusted for length; and arm muscle area adjusted for arm length
- Neonatal body composition (via PEA POD®) including fat mass and lean mass adjusted for length and fat mass adjusted for lean mass
- Gestational age at birth
- LGA, by customised [73] and population [72] references.
- Small for gestational age (SGA, <10^th^ centile), by customised [73] and population [72] references.
- Admission to neonatal care unit (and reason)
- Neonatal composite morbidity, including birth trauma (fracture, brachial plexus injury, cephalohaematoma, subgaleal haematoma), hypoxic ischaemic encephalopathy, sepsis, respiratory distress requiring continuous positive airway pressure support, hypoglycaemia requiring dextrose treatment (buccal or intravenous)
- Initiation and establishment of breast feeding, including feeding in first two postnatal weeks (collected by phone call at 6 weeks and questionnaires)
- Infant anthropometry and body composition at 5 months of age, as detailed above
- Infant feeding over first 5 months (breast feeding, formula use, complementary feeding with solids)
- Feeding behaviour as assessed by BEBQ scores [69]
- Infant Nutritional intake at 5 months, estimated from a four day food frequency questionnaire

Other secondary outcomes include:

- Attendance at study visits
- Adherence to probiotic/placebo regime
- Cost effectiveness of the intervention

### Sample size and power calculation

A total of 220 participants will be recruited. With 80% power and 100 subjects remaining in each main intervention group (allowing 10% lost to follow-up) we can detect: 25% reduction in excessive GWG from 80% to 60% (based on an 80% rate of excess weight gain in obese participants in the SCOPE study) [16] and 227 g difference in mean birthweight (based on CMH data; mean = 3,638, SD = 521). To allow for the two primary outcomes an alpha of 0.025 has been used for the power calculations (Bonferroni approach).

### Statistical analysis plan

Analyses will follow the principle of intention-to-treat. Participants will be analysed according to the assigned treatment group at randomisation. Statistical models will adjust for the key randomisation stratification variable, BMI at recruitment.

Binary endpoints will be analysed using logistic regression to estimate odds ratios for each of the interventions (dietary intervention and probiotics). Continuous outcomes will be modelled using generalised linear models to estimate any changes in outcomes with the interventions (dietary intervention and probiotics) compared to their respective control groups.

Primary analyses will report marginal effects for each randomised exposure, with adjustment for co-intervention, BMI at recruitment (randomisation stratification variable), ethnicity and sex (infant outcomes). We will also test for interactions between the main effects (primary outcomes only), though this pilot trial has been powered only for the main effects.

Sensitivity analyses will be carried out according to compliance with the study interventions (primary outcomes); specifically these will be:

- Those women who reported taking their probiotics most of the time at all time points (forgot to take 1–3 per month, or greater than 1 per week but not most days at 28 weeks, 36 weeks and birth visits).
- Those that attended at least 3 out of the 4 dietary education sessions.

Analyses of secondary outcomes involving maternal data collected at recruitment, 36 weeks’ and 5 months’ will be analysed using mixed methods to allow for the repeated measures of these outcomes over time. Measures of infant body composition (whole-body fat and fat-free mass) will be adjusted by infant sex and ethnicity, and skinfold thickness and arm muscle area will additionally be adjusted for infant length. For birth data, analyses will be adjusted for gestational age at birth (weeks). We will explore for an interaction between intervention effect and infant sex.

For the primary outcomes a two-sided alpha level <0.025 will indicate statistical significance (Bonferroni adjustment). For secondary outcomes, a two-sided alpha level <0.05 will indicate statistical significance.

Depending on the proportion and the pattern of missing data, multiple imputation may be used to impute missing data for some exploratory variables (secondary outcomes).

### Safety monitoring

The research team will oversee and manage the project. A Trial Steering Committee has been established that contains a representative group of the named investigators who are responsible for the conduct of the trial and will follow CONSORT guidelines [46]. An independent committee (with no conflict of interest) has been appointed with established terms of reference to serve as the Data Safety and Monitoring Committee (DSMC). The DSMC includes experts in the fields of obstetrics, neonatology and epidemiology/statistics with experience in perinatal trials. They will assess the progress of the trial and review reports of serious adverse events and adverse events.

Serious adverse events are defined as maternal death, maternal admission to intensive care unit, fetal death, neonatal death or death up to primary hospital discharge, stage 2–3 neonatal encephalopathy, and any other serious adverse event that the principal or local lead investigator believes should be referred for independent review by the DSMC.

The DSMC will classify serious adverse events as to the likelihood of a causative association with the study intervention: no, unlikely, possible, or probable. The DSMC can request unblinding if there is any safety consideration related to the probiotic/placebo intervention.

### Data management

Data will be collected in a timely fashion and entered directly into the web-based REDCap™ [74] study database using mobile tablets. Data are actively monitored by a data monitor following the Data Resolution Workflow produced by REDCap™ [74]. Electronic case record forms (CRFs) will be identified only by a study identification number (ID), with consent forms and identifying information stored in a separate, restricted database. In rare cases, when paper CRFs are used they will be coded using the study ID number, ensuring confidentiality of participants. All paper CRFs will be stored securely in locked cabinets for 10 years and access to all electronic data will be password protected and restricted to researchers directly involved with the study.

No interim analysis is planned.

## Discussion

This two by two factorial design randomised controlled demonstration trial aims to reduce excessive GWG and optimise infant birthweight in a multi-ethnic sample of obese pregnant women. This RCT will inform whether a multifaceted dietary intervention and or probiotic treatment, are feasible interventions for obese pregnant women in the muti-ethnic CMH region. If successful, either one or both of the interventions are applicable to future clinical practice. It may also inform the design of a larger-scale intervention study for obese pregnant women that is powered to investigate pregnancy complications.

### Dissemination policy

We will publish the results of HUMBA in scientific journals and present the results at relevant conferences locally and internationally. Our publications will also be available on the HUMBA study website, and will be shared by email with participants who have consented to receive a copy of the findings. Our multidisciplinary team of investigators will disseminate research findings widely via their clinical networks, including giving seminars and presentations to health professionals and community groups. We will utilise newsletters of relevant professional bodies to further disseminate research findings. Findings from HUMBA will also be incorporated into the relevant Cochrane systematic reviews thereby increasing dissemination both locally and internationally.

## Abbreviations

BEBQ: Baby Eating Behaviour Questionnaire
BMI: Body mass index
BP: Blood pressure
CHW: Community health worker
CMH: Counties Manukau Health
CRF: Case report form
DSMC: Data safety and monitoring committee
EPDS: Edinburgh Postnatal Depression Scale
GDM: Gestational diabetes mellitus
GWG: Gestational weight gain
HUMBA: Healthy mums and babies
IADPSG: International Association of the Diabetes and Pregnancy Study Groups
LGA: Large for gestational age
NZSSD: New Zealand Society for the Study of Diabetes
OGTT: Oral glucose tolerance test
RCOG: Royal College of Obstetricians and Gynaecologists
RCT: Randomised controlled trial
SF-12: 12-item Short-Form Health Survey
STAI: State-Trait Anxiety Inventory
