# Correction to: A randomised controlled demonstration trial of multifaceted nutritional intervention and or probiotics: the healthy mums and babies (HUMBA) trial

**DOI:** 10.1186/s12884-018-1792-3

**Published:** 2018-05-04

**Authors:** Karaponi Okesene-Gafa, Minglan Li, Rennae S. Taylor, John M. D. Thompson, Caroline A. Crowther, Christopher J. D. McKinlay, Lesley M. E. McCowan

**Affiliations:** 1 Department of Obstetrics and Gynaecology, Faculty of Medical and Health Science, The University of Auckland, Auckland, New Zealand; 2 South Auckland Clinical School, Faculty of Medical and Health Science, The University of Auckland, Auckland, New Zealand; 3 The Liggins Institute, The University of Auckland, Auckland, New Zealand; 4 Department of Paediatrics: Child and Youth Health, Faculty of Medical and Health Sciences, The University of Auckland, Auckland, New Zealand

## Correction

In our ‘Primary Outcomes’ we made a typographical error [1]; the mean weekly weight gain should read >0.27 kg instead of >0.22 kg. The ‘Primary Outcomes’ should read as follows:

## Primary outcomes

The primary maternal outcome is the proportion of women with excessive GWG, defined as mean weekly weight gain >0.27 kg between recruitment and 36 weeks (or weight at the closest gestation to 36 weeks’ if 36 week weight is unavailable) [15].

The primary infant outcome is infant birthweight.
